# Interventions to Improve Health among Reproductive-Age Women of Low Health Literacy: A Systematic Review

**DOI:** 10.3390/ijerph17207405

**Published:** 2020-10-12

**Authors:** Rafael Vila-Candel, Francisco Miguel Martínez-Arnau, Juan María de la Cámara-de las Heras, Enrique Castro-Sánchez, Pilar Pérez-Ros

**Affiliations:** 1 Department of Obstetrics and Gynaecology, Hospital Universitario de la Ribera, 46600 Valencia, Spain; rafael.vila@uv.es; 2 Department of Nursing and Podiatry, Faculty of Nursing and Podiatry, Universitat de València, 46010 Valencia, Spain; maria.p.perez-ros@uv.es; 3 Department of Physiotherapy, Universitat de València, 46010 Valencia, Spain; 4 Library Department, Hospital Universitario de la Ribera, FISABIO. Crta. Corbera km 1, 46600 Valencia, Spain; delacamara_jua@gva.es; 5 School of Health Sciences, University of London, London EC1V, UK; Enrique.Castro-Sanchez@city.ac.uk

**Keywords:** health literacy, numeracy, reading ability, reading skill, pregnant women, intervention

## Abstract

*Background*: Limited or low health literacy (HL) has been associated with poor health outcomes, including inadequate self-caring and preventive behaviors. A few studies have systematically summarized the effect of interventions to improve reproductive health and care in women with insufficient HL. The main objective of the study was to investigate health care promotion interventions and examine their effectiveness on women with inadequate HL through a systematic review of randomized controlled trials (RCT). *Methods*: RCTs and quasi-experimental studies that assessed HL interventions to improve reproductive health of women with low HL were included. The study protocol was registered with PROSPERO (CRD42020137059). *Results:* Of the 292 records initially identified, a total of 6 articles were included for review. Five different HL screening tools were used. Four different interventions were included: educational intervention, communication skills, a multimedia interactive tool, and text adaptation to enhance reading comprehension. Not enough research practice has been conducted on the influence of interventions on HL, and thus, it is difficult to implement evidence-based interventions. *Conclusions*: Interventions aiming to benefit and improve HL should consider the complex web of intersectional determinants that end up shaping the opportunities of women to make optimal decisions regarding their health and care, and which may require attention to much more than clinical or service delivery factors.

## 1. Background

Health literacy (HL) relates to a person’s knowledge and skills in decision-making in a medical and social context necessary for meeting the complex health demands of modern society [1]. Essential HL skills include reading, writing, numeracy, and searching for information [2], using multimedia technologies and solving problems, all of which are essentially personal and social skills for navigating the health system [3]. HL it is considered one of the most important factors and determinants of individual health and health service use [4].

A strong heterogeneity in defining and measuring HL between women and men has been reported [5]. There have been different studies validating the correlation between poor or low HL in women and poor health outcomes [6,7]. A woman’s level of HL has the potential of impacting the health outcome of her entire family [8,9].

Currently, various standardized and validated tools have been proposed for assessing HL, but, to date, none of them are considered the “gold standard” [10,11]. There is currently controversy regarding the routine use of HL screening for clinical purposes, although most disagreement is focused on its use on patients or on specific individuals rather than the overall population [12]. Some authors recommend considering the entire population as having a low HL level [9], claiming that routine screening of HL lacks benefits and could have undesired effects. On the other hand, different professional organizations promote HL screening to reach the largest possible population and provide understandable and accessible information, regardless of the level of HL [13]. 

Inadequate HL serves as a potential mediator of health disparities, and has been related to level of education (less than high school), low socioeconomic status, Hispanic ethnicity, Black race, and older age [14]. Limited or low HL has been associated with poor health outcomes, including inadequate self-caring and preventive behaviors [15].

Different interventions have been already designed to improve the outcomes and experience of patients with low HL in relation to health problems or pathologies surrounding maternal health: gestational weight gain [16], diabetes mellitus [17], breastfeeding promotion [18]; Zika virus [19], medication errors [20], breast cancer [21], and down syndrome screening [22]. These interventions have focused on increasing health-related knowledge in these processes, increasing patients’ comprehension, improving adherence to treatment, or improving patient–provider communication. Furthermore, these interventions include a variety of approaches and components, from face-to-face communication to personalized teaching classes with in-person counselling, and interactive or technology-assisted or education multi-media with interpersonal interactions.

Overall, there has been relatively little systematic research comparing the effectiveness of interventions, specifically with regards to any relationships between HL and health outcomes [23,24]. More evidence is needed to identify the optimal way to design interventions to decrease health disparities in women with low HL. Among these areas of research, further validation of the benefits of clearer health communication on health outcomes, assessment of mediators other than communication in the pathway between literacy and poor health outcomes, and further assessment of the homogeneity of persons with low HL are required to develop appropriate interventions for them [25]. Thus, the objectives of this study were to investigate the breadth, depth and quality of the literature relating to the following questions:

1. What screening tools have been used to measure the level of health literacy of women or pregnant women?
2. What interventions and characteristics were carried out in this group?
3. What is the effect of interventions carried out on women with limited or inadequate health literacy, including pregnant women, in order to improve health care?

## 2. Methods

The PICO format (population/intervention/comparation/outcomes) was used to prepare the research question, as shown in Table 1 [26]. To address these questions, a systematic review of published research was conducted between October to November 2019 following guidelines outlined in the *Cochrane Handbook for Systematic Reviews of Interventions.* The details of the protocol for this systematic review were registered on PROSPERO ID: CRD42020137059.

### 2.1. Search Strategy

Studies were identified from MEDLINE (from OVID SP), the Cumulative Index to Nursing and Allied Health Literature (CINAHL from EBSCO), Embase (from OVID SP), and the Cochrane Database of Systematic Reviews (from OVID SP). Each database was searched using the search terms shows in Table 1 as a single search term or in combination using Medical Subjects Heading (MeSH) terms with the Boolean operators AND/OR [27].

The search for unpublished studies included an electronic search of trial records: current controlled trials (http://www.controlled-trials.com), the National Institute of Clinical Health Databases (https://clinicaltrials.gov), the Universal Index of Doctoral Dissertations in Progress, Mednar, review of the grey literature and Google search.

### 2.2. Inclusion and Exclusion Criteria

The inclusion criteria were as follows: (a) peer reviewed; (b) studies on interventions reported as specifically designed to mitigate the effects of low HL in women or pregnant women; (c) articles that measured HL using a previously validated HL assessment; (d) outcome measures provided evidence on the relationship between HL and reproductive health outcomes or related knowledge or behaviors; (e) studies published in English or Spanish languages; (f) studies from January 1995 to November 2019; (g) randomized clinical trials and quasi-experimental studies conducted with comparison groups with level of HL.

### 2.3. Data Extraction and Quality Assessment

The authors created a data extraction form tailored to this investigation using the guidelines outlined in the Cochrane Handbook for Systematic Reviews of Interventions [28]. Two independent authors reviewed the papers. The data extraction procedure was conducted in two phases: (1) by title and abstract, and (2) by full text. Following the assessment of title and abstract, the primary reviewer (RVC) and secondary reviewer (FMMA) performed the full-text evaluation. A third reviewer (PPR) acted to resolve any disagreements.

The first and second authors thoroughly reviewed each study and extracted the main data: study design, sample characteristics, sample size, location of the study, the HL screening tool, health intervention characteristics, HL measures, outcome measures, and reported results.

Any coding discrepancies between the two authors were resolved through subsequent review. Abstracted data were then compiled, reviewed, and summarized in table format by one study author (RVC). After determining article inclusion, one reviewer entered data about each study onto the evidence tables, with the second author checking and validating the information for accuracy.

### 2.4. Assessment of Risk of Bias within Selected Studies

Two reviewers independently rated the quality of studies using the Cochrane protocol that assesses bias (RoB2). The RoB2 tool comprises seven domains: random sequence generation; allocation concealment; blinding of participants and personnel; blinding of outcome assessment; incomplete outcome data; selective reporting; and other sources of bias. Each evaluation was classified by two independent authors to a high risk of bias, a low risk of bias, or an unclear bias.

### 2.5. Data Synthesis and Analysis

As the number of studies with similar outcomes was modest, and the interventions heterogeneous, a narrative synthesis was agreed upon. However, to central tendency and dispersion values contributed by the authors at the pre- and post-moment, a percentage of change in the main variables was calculated to facilitate comparison between the groups.

## 3. Results

### 3.1. Characteristics of Clinical Trials

**Figure 1:**
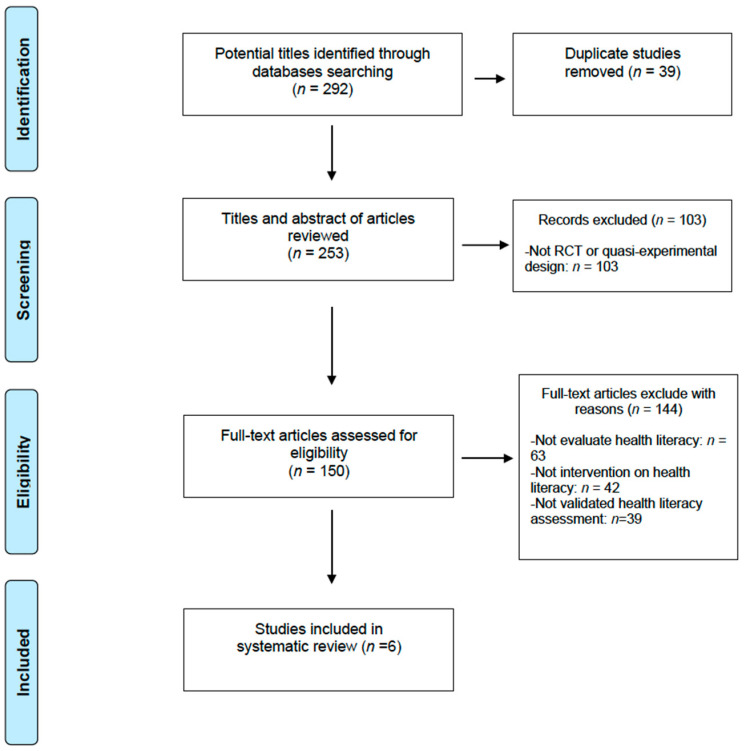
Preferred reporting items for systematic reviews and meta-analyses (PRISMA) flow diagram.

The search retrieved 292 articles. After the study selection process, six articles were included in the analysis. The full study selection process is presented in Figure 1 as per recommended preferred reporting items for systematic reviews and meta-analyses (PRISMA) criteria [29].

Details of the study characteristics are presented in Table 2. The included studies were published between 2011 and 2019. Six studies from three countries met the criteria: The United States of America (*n* = 3), Iran (*n* = 2) and Australia (*n* = 1). Five studies applied an RCT design, while only one used quasi-experimental design. The sample sizes ranged from 80 to 1126 patients.

The themes in which HL were evaluated, including self-care in pregnant women, teach-back in telehealth services for women, gestational diabetes in pregnant women, preterm prevention in pregnant women, prenatal genetic information in pregnant women, and informed consent in tubal sterilization.

### 3.2. Results of Health Literacy Screening Tools

Table 2 presents the five HL screening tools used. Two studies (33.3%) used the Single Item Literacy Screener (SILS) test. The remaining tools were the Maternal Health Literacy and Pregnancy Outcome Questionnaire (MHLAPQ), the Iranian Health Literacy Questionnaire (IHLQ), the Short version of the Test of Functional Health Literacy in Adults (S-TOFHLA), and the Rapid Estimate of Adult Literacy in Medicine (REALM).

### 3.3. Results of Interventions to Support Women with Low Health Literacy

Table 3 presents the results of interventions for six studies. Four different components were included: educational sessions, communication skills by telephone, a multimedia interactive tool, and text adaptation to enhance reading comprehension. Three studies used educational intervention (50.0%), and the others used communication skills by telephone (16.7%), a multimedia interactive tool (16.7%), and text adaptation (16.7%).

### 3.4. Quality of Evidence

**Figure 2:**
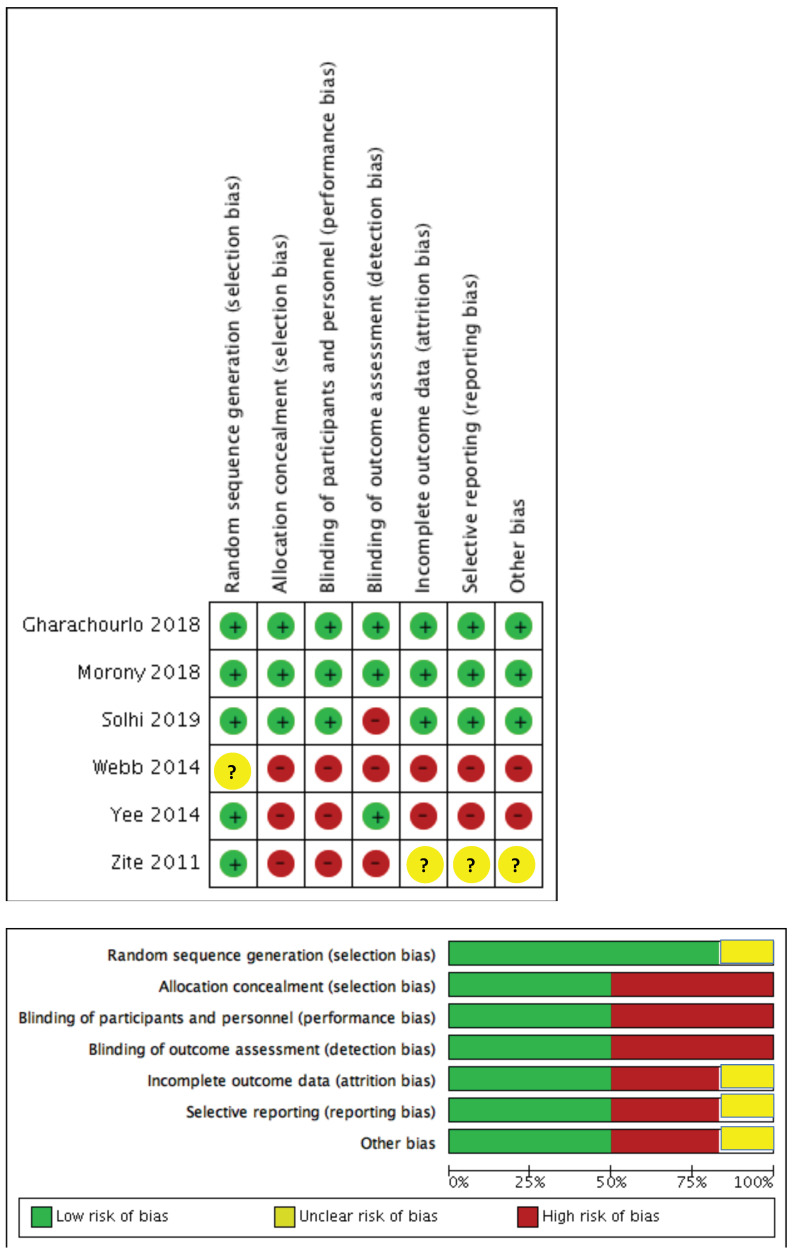
Risk of bias of the included studies.

Figure 2 presents information about the risk of bias of the six selected studies. Two studies (33.3%) satisfied the seven item of risk bias, whereas one study (16.6%) satisfied six items of risk bias. Three studies (50.0%) satisfied less than two item of risk bias. Five studies (83.3%) were deemed as having low risk allocation, whereas in the remaining one (16.6%), the allocation sequence was not reported. Allocation concealment was identified in three of these studies (50.0%). Half of selected studies (50.0%) did not blind the experimental group. Three studies (50.0%) had low risk of attrition bias; however, attrition rates were not included in one study (16.6%). Likewise, three studies (50.0%) were deemed to have a high risk of reporting bias as they did not provide key outcome variables.

### 3.5. Results of Effects of Interventions for Women with Low Health Literacy

The study of Solhi et al. [30] explored the effect of HL education on various self-care outcomes for pregnant women in the Islamic Republic of Iran.

The intervention included an education package on HL and self-care during pregnancy delivered over four 45-min sessions with lectures, counselling, interactive group discussions and practical exercises, as well as educational materials.

Data collected from the participants included dedicated self-care and health literacy (MHLAPQ) questionnaires focused on the pregnancy period. The validity and reliability of both questionnaires was confirmed with content validity ratio (CVR) and content validity index (CVI).

Before the intervention, there was no significant difference between the groups in terms of the mean values of computational comprehension (*p* = 0.59), reading comprehension (*p* = 0.97), behavior (*p* = 0.7), and total HL (*p* = 0.62). However, there were significant differences in all these variables at 1- and 2-months post-intervention (*p* < 0.001). The self-care and HL scores pre- and post-intervention were also significantly different, with greater increases in the intervention group and with an increase in the intervention arm (IA) of 29.5% per month and 31.4% at 2 months, and by 1.6% per month and 3.9% at 2 months in the control arm (*p* < 0.001).

The study of Morony et al. [31] aimed to implement the “teach-back” technique (wherein patients are asked to repeat, in their own words, the information they had just received) in a telephone service providing information and advice for pregnancy and parenting of young children, and evaluate the impact on caller ratings of information they received as well as the overall experience of the call (Australian national pregnancy and parenting telephone helpline). This study aimed to mitigate the service gaps experienced by persons with low HL when accessing telephone-based health services in view of their difficulties to ask questions or indicate when they do not understand.

The intervention involved a single 2-h group “teach-back” training session in different groups of nurses who were trained in theoretical and practical communication skills, combined with self-reflection on such communication following each call and shift. The control arm (users) received usual care.

The HL level was evaluated by the Single Item Literacy Screener (SILS). A total of 87% of callers to the telephone helpline were female, of whom 13% had an inadequate HL. Among the nurses, 83% had an average of 15 years’ experience on maternal and child health.

There was no independent effect of “teach-back” on having sufficient information to manage health (*p* = 0.37). However, the authors identified that “teach-back” could increase perceptions relevant to self-efficacy in callers with inadequate HL (confidence, actionability and share decision-making). Of note, the statistical level of significance was set at *p* < 0.10 by the authors.

Persons with lower HL in particular appeared to benefit from “teach-back”, without any evidence of negative or undesired impacts on caller outcomes, including satisfaction.

The aim of the study of Gharachoulo et al. [32] was to investigate the effect of an HL approach to counselling on the lifestyle of women with gestational diabetes.

Participants allocated to the intervention arm were offered counselling about pregnancy care and HL-tailored advice about lifestyle. The control arm (CA) only received counselling about standard pregnancy care. Both groups completed the Iranian Health Literacy Questionnaire (IHLQ) and Lifestyle Questionnaire (LSQ) at the start, end and three weeks post-sessions. The authors did not report on the prevalence of inadequate HL. Before the intervention, the two groups obtained comparable HL scores. The mean score of HL, however, increased more in the intervention arm immediately and at three weeks post-intervention compared to the control group (immediately post-intervention: IA: + 44.7% vs. CA: +12.5%; 3 weeks post-intervention: IA: +32.7% vs. CA: 8.7%; *p* < 0.001).

The findings suggest the effect and role of counselling and the counsellor midwife in improving HL in mothers with gestational diabetes, as well as that education and counselling increased HL in diabetics with any level of HL. On the other hand, there were no significant differences between the two groups before counselling in terms of the score of lifestyles.

The main purpose of the randomized clinical trial conducted by Webb et al. [33] was to evaluate the performance of a bundle of evidence-informed risk-reduction strategies applied during the inter-conception period to reduce the risk of a subsequent preterm birth among women who had delivered a preterm infant. The Short Test of Functional Health Literacy in Adults (S-TOFHLA) was used.

All participants were assessed at 1, 6, 12, 18 and 24 months postpartum. The IA were regularly assessed for the presence of the pre-specified risk factors, and participants were invited benefit from state-of-the-art treatment and services offered as part of the protocol. Compared to White (8.7%) women, HL levels were lower in Black (24.0%) and Hispanic (24.7%) participants.

About 40% of women with low HL received any related services, and fewer than half of women with periodontal disease had follow-up consultations.

The aim of the study by Yee et al. [34] was to determine whether an interactive computer program could improve patient knowledge regarding genetic screening and diagnostic concepts. The IA received an interactive, computer-delivered, 3D visualization of the body, coupled with educational packages on essential prenatal testing concepts. Women in the standard care arm (CA) were only offered routine antenatal counselling during their clinic appointments. HL and computer literacy were assessed via the Rapid Estimate of Adult Literacy in Medicine (REALM) and eHealth Literacy Scale.

The proportion of women with limited HL was 43%. eHealth Literacy Scale scores on each group were comparable. Pregnant women allocated to an interactive, computer-delivered tool, with information on prenatal genetic screening knowledge increasing post-test when compared to women who were allocated to routine counselling. In this study, women exposed to the computer-delivered intervention improved their scores as much as women in the standard care group, regardless of level of education, health literacy, or e-HL, however, our analysis obtains contradictory results (23 days post-intervention: IA: −12.7% vs. CA: +8.0; *p* = 0.001). Equally, the benefit was independent of women’s familiarity with electronic resources.

The aim of the study conducted by Zite et al. [35] was estimate whether the Medicaid-Title XIX Sterilization Consent Form (SCF) format—“standard” (high school reading level) compared with “low-literacy” (6th grade reading level)—led to increased understanding of tubal sterilization among women.

The HL level was evaluated by the Single Item Literacy Screener (SILS). Patients were given relevant informed content in two formats, the standard version and a low-literacy version. Participants were then asked about the content of both informed consents. Participants were categorized as having limited (zero to three correct responses) or adequate (four or more correct responses) sterilization-related knowledge based according to their responses to these items. The prevalence of women with low HL was 48.5–50.0%. The findings suggest that, without additional counselling or clarification by a clinician and compared with the standard consent form, the low-literacy version increased women’s understanding of the clinical procedure (*p* < 0.01).

## 4. Discussion

To our knowledge, this is the first attempt to systematically summarize the effect of interventions to improve health and care in women with insufficient HL. The previous systematic reviews of the relationship between HL and women’s reproductive health [6,36] have identified a limited number of RCTs, and many lacked clarities as to whether the interventions were an HL intervention consistent with the definition [24]. A previous systematic review of the effect of HL interventions on pregnancy outcomes similarly identified a limited number of RCTs, with only 2 of the 13 assed by an HL screening tool in order to explore the impact of HL interventions on health outcomes [24]. This review identified three new RCTs from the previous, indicating that there is possibly greater interest in the topic; however, identified HL interventions have important research gaps that have to be improved in future research.

The distribution of countries in our review was perhaps surprising, with hardly any research conducted in low- and middle- income countries. Perinatal health is a universal component of health services worldwide. HL is a construct that includes socioeconomic components (educational level, social level and economic income) [1,4]. Improvement interventions could be direct (providing additional information, personalized and adapted to the HL level) or indirect (improving the socioeconomic components of women) and are likely to influence their HL level and expand the range of research interventions across both domains. Despite such ubiquity and the importance of optimal care for women and pregnant women, gaps remain in the evidence regarding the influence of HL and interventions aiming to mitigate such deficits. It is also clear that there is no single intervention that will have a great impact on these outcomes. Instead, a combination of interventions applied at various times during pregnancy, the post-partum period or in the neonatal period are needed to improve the health care of women. Our review identified a modest and heterogeneous sample of studies centered on women’s HL, mostly interested in the effect of HL on pregnancy. HL levels of pregnant women can help them to detect and understand danger signs in their pregnancy processes, to take adequate care and to adhere to the providers’ advice during the antenatal program [37]. Although it is true that women with low HL have less knowledge about the prenatal screening test for birth defects, we observed that in this case, the control arm obtained an improvement when compared to the intervention arm, but this was not explained by the authors [34]. Possibly, these differences between the groups already existed from the beginning.

HL is higher in people with a higher educational level [38]. However, the level of education is not a definitive indicator of HL. Based on the findings of our study, even if women reported a higher educational level, their HL levels should not be assumed to be adequate and should be examined carefully by health care providers.

The use of a variety of HL screening tools is not unexpected, but it is surprising that none of the studies use more modern tools—HLQ [39] and HLS-EU-Q47 [40], for example, being among the most prominent.

The concentration of the selected studies in the last 5 years is unanticipated, despite the interest and support shown by WHO for information offered by healthcare professionals to patients to be suitably tailored to improve outcomes. The aim of such information provision is not just so that the person knows more about their health problem, but that they also gain skills in identifying information that is appropriate and accurate, allowing them to make decisions about care based on such information, their settings and skills [11,41].

Overall, the quality of the studies was medium/poor, and the interventions achieved results constrained by limitations. Regarding the themes, these were mostly pathology-focused, with a couple centered on preventive self-efficacy. None of the studies related to classic public health issues such as vaccination adoption during pregnancy, a reduction in the number of caesarean sections, breastfeeding or tobacco consumption during pregnancy. Future studies should evaluate interventions within these remits.

Whilst this review did not aim to include evidence of economic outcomes of the interventions explored, such evidence is essential before considering the widespread implementation of measures to mitigate the impact of low HL. Although the economic consequences of such low HL are well recognized in relation to poorer health status, lack of knowledge about medical care, lack of use of preventive services, increased hospitalizations and health care costs, few studies on the other hand have provided any indication of costs related to HL interventions [42,43].

Finally, it may be beneficial for other interventions to explore how the HL of women, partners and support networks interact in dyads as seen in other health problems [44], as well as the potential of measures that increase “collective” or community HL.

Among the limitations of this study, we can highlight that the studies included in the review presented different research designs, which together with the use of different methods of assessing HL due to the lack of standardization on the subject, make the comparison of them more complicated and limit the interpretation of quantifiable effects. However, one finding is shared by the different studies, that is, the importance of increasing HL in clinical nursing education as a key point for patient decision making. The inclusion of studies with different sample sizes and quality standards may have affected the homogeneity of the results obtained.

Finally, although relevant terms were added in the search items, the HL search strategies were not strictly followed, and this together with the exclusion of papers in languages other than those detailed in the inclusion criteria may have caused some bias in the selection of the articles.

HL, or the skills needed to function in the health care environment, is recognized as a mediator of health disparities [45]. Inadequate HL has a clear association with poor health outcomes, inadequate utilization of health care services, and poor health knowledge [15]. Our finding was the focus on developing an intervention appropriate for women with limited HL, because this population could benefit from added health education. Furthermore, educational interventions that benefit patients in low literacy populations typically benefit individuals in higher literacy groups as well [46]. Inadequate HL is a common public health problem, even in populations with high amounts of formal education [47]. As shown here, education alone is inadequate to prepare women for the complex clinical counselling along the pathway of women’s care and health. New studies with multidimensional methodologies are needed to identify the best strategy for each of the health processes that we are interested in addressing.

## 5. Conclusions

Health literacy is a crucial factor contributing to women’s self-efficacy and optimal care. Interventions aiming to benefit and improve health literacy should consider the complex web of intersectional determinants that end up shaping the opportunities of women to make ideal decisions about their health and care, and which may require attention to much more than clinical or service delivery factors. Our review has highlighted the size of the task ahead. More research is needed in order to improve this knowledge and its relation to other outcomes in women or pregnant women.

## Figures and Tables

**Table 1:** PICO strategy: category, definition, and search terms in databases.

Category	Definition	Search Terms for Embase Pregnancy	Search Terms for Medline Pregnancy	Search Terms for CINAHL
Population	Women or pregnant women	exp PREGNANCY/or exp WOMEN/.ti,kw.	exp Pregnancy/or Women	prepregnancy OR pre pregnancy OR pregnant preconception* OR pre conception* OR periconception* OR women
Intervention	Interventions that authors report are designed specifically to mitigate the effects of low health literacy. Uses single or multiple literacy-directed strategies.	(“poor health literacy” or “health literacy” or “literacy, health”).mp. or exp “health literacy”/use oemezd or exp “Health Literacy”/use medall	health literacy OR literacy OR numeracy OR reading ability OR reading skills OR poor health literacy OR litercy, health	health literacy OR poor health literacy OR literacy, health
Comparisons	Not applicable			
Outcomes	Health care, obstetric care, reproductive care	exp OBSTETRIC PROCEDURE/or exp BREAST FEEDING/or exp BREAST FEEDING EDUCATION/or exp BIRTH/or exp CHILDBIRTH/or CHILDBIRTH EDUCATION/or LABOR PAIN/or (ante natal or antenatal* or pre natal* or prenatal* or puerper* or postnatal* or postpartum or post partum or post natal* or peripartum or peri partum or prepregnancy or pre pregnancy or preconception* or pre conception* or periconception* or peri conception* or or (pregnancy or pregnancies or pregnant)((preterm or premature) and (labor or labour)) or eclamp* or preeclamp* or pre eclamp* or amniocentes* or chorion* vill* or breastfe* or breast fe* or lactation* or cesarean or caesarean or cesarian or caesarian or cesarien or caesarien or newborn* or new born* or tocoly* or fetal or foetal or fetus or foetus or miscarriage*) or care or health care.ti,ab,kw.	exp Pregnancy Complications/or exp Obstetrics/or exp Breast Feeding/or exp Prenatal Education/or exp Labor Pain/or (breast-feeding education or parturition or ante natal antenatal* or pre natal* or prenatal* or puerper* or postnatal* or postpartum or post partum or post natal* or peripartum or peri partum or prepregnancy or pre pregnancy or preconception* or pre conception* or periconception* or peri conception* or ((preterm or premature) and (labor or labour)) or eclamp* or preeclamp* or pre eclamp* or amniocentes* or chorion* vill* or breastfe* or breast fe* or lactation* or cesarean or caesarean or cesarian or caesarian or cesarien or caesarien or newborn* or new born* or tocoly* or fetal or foetal or fetus or foetus or miscarriage* or pregnancy or pregnancies or pregnant) or care or health care.ti,ab,kf.	(antenatal* OR prenatal* OR puerper* OR postnatal* OR postpartum* OR post partum OR post natal* OR peripartum OR peri partum) OR care OR health care OR

PICO: population/intervention/comparation/outcomes; CINAHL: Cumulative Index to Nursing and Allied Health Literature.

**Table 2:** Characteristics of the studies included in the review.

Author	Design	Study Period	*n*	Theme	Age	Country	HL Tool
Solhi et al., 2019 [30]	RCT	Jan to June 2016	80	Self-care in pregnant women	>18 years	Iran	MHLAPQ
Morony et al., 2018 [31]	QES	July to Oct 2018	637 callers and 18 nurses	Teach-back in telehealth service	31.3 ± 6.5	Australia	SILS
Gharauchourlo et al., 2018 [32]	RCT	6 weeks (1.5-h session once a week)	84	Pregnant women with gestational diabetes	IG: 31.5 ± 4.4 CG: 30.8 ± 3.8 *p* = 0.734	Iran	IHLQ
Webb et al., 2014 [33]	RCT	Sep 2004 to Aug 2008	1126	Preterm prevention project	25.6 ± 6.6	USA	S-TOFHLA
Yee et al., 2014 [34]	RCT	Aug 2010 to March 2011	150	Prenatal genetic information	26.6 ± 5.3	USA	REALM
Zite et al., 2011 [35]	RCT	May to July 2010	203	Informed consent in tubal sterilization	21–45 years	USA	SILS

HL: Health Literacy; RCT: randomized clinical trial; MHLAPQ: Maternal Health Literacy and Pregnancy Outcome Questionnaire; QES: quasi-experimental study; SILS: Single Item Literacy Screener Test; IG: intervention group; CG: control group; IHLQ: Iranian Health Literacy Questionnaire; S-TOFHLA: Short version of the Test of Functional Health Literacy in Adults; REALM: Rapid Estimate of Adult Literacy in Medicine.

**Table 3:** Description of articles that explored health literacy.

Author	Intervention	Health Interventions	Outcome 1	Intervention Group Average and SD	Control Group Average and SD	Other Reported Findings	Outcome 2	Intervention Group Average and SD	Control Group Average and SD
Solhi et al., 2019 [30]	Control group (*n* = 40) received the routine educational program. The intervention group (*n* = 40) received the routine educational program and additionally followed the educational intervention sessions.	Educational intervention sessions of 45 min each in the form of lectures, group discussion, question and answer session, counselling, practical exercises, and educational materials (e.g., booklets and films about pregnancy).	Determine the effect of health literacy education on self-care in pregnant women.	Before intervention 30.9 ± 5.3 1 month after intervention 40.0 ± 3.5 2 months after intervention 40.6 ± 3.1	Before intervention 30.4 ± 4.9 1 month after intervention 30.9 ± 4.6 2 months after intervention 31.6 ± 4.6	Before intervention *p* = 0.62 1 month after intervention *p* < 0.001 2 months after intervention *p* < 0.001	Self-care questionnaire	Before intervention 62.9 ± 6.3 1 month after intervention 76.8 ± 4.3 2 months after intervention 78.0 ± 3.9	Before intervention 62.6 ± 6.5 1 month after intervention 65.0 ± 6.2 2 months after intervention 66.0 ± 6.7
Morony et al., 2018 [31]	Training in theory and skills for using teach-back was a 2-h “communication skills” workshop. For the duration of the study, nurses were encouraged to reflect after each call on how effectively they communicated and how well the caller understood. Caller outcomes were assessed in a single telephone survey conducted by population research laboratory PRL approximately one week following initial contact.	Handling of telephone calls by means of the teach-back method.	Evaluate the impact of teach-back on communication quality in a national telephone-based telehealth service for callers varying in health literacy.	45.5% (*n* = 116) in highest category	40.2% (*n* = 150) in highest category	Odd ratio OR= 0.77 (95% CI 0.44–1.37); *p* = 0.37	Satisfaction of callers and nurses	72.3% (*n* = 188) in highest category	70.7% (*n* = 266) in highest category
Gharauchourlo et al., 2018 [32]	Six weeks (1.5 h sessions once a week) IG (*n* = 50): received counselling on routine pregnancy care and a health literacy approach to counselling for modifying lifestyle. CG (*n* = 50): received counselling on routine pregnancy care as well as a training package containing all the subjects discussed in the intervention group.	Educational intervention with counselling on routine pregnancy care and a health literacy approach to counselling for modifying lifestyle.	Investigate the effect of a health literacy approach to counselling on the lifestyle of women with gestational diabetes.	HL: Before intervention 9.95 ± 2.52 After intervention 14.4 ± 1.3 3 weeks after intervention 13.2 ± 1.8	HL: Before intervention 10.4 ± 2.1 After intervention 11.7 ± 1.9 3 weeks after intervention 11.3 ± 1.9	*p* < 0.001; F = 278.7	Lifestyle Questionnaire (LSQ)	Before intervention 144.7 ± 21.5 After intervention 175.6 ± 12.8 3 weeks after intervention 184.0 ± 12.2	Before intervention 143.5 ± 19.9 After intervention 151.3 ± 18.3 3 weeks after intervention 153.4 ± 16.6
Webb et al., 2014 [33]	Women randomized into the treatment group (*n* = 565) were regularly assessed for the presence of the pre-specified risk factors and invited to avail themselves of the state-of-the-art treatment and services offered as part of the Philadelphia Collaborative Preterm Prevention project PCPPP protocol. Women who were randomized into the control group (*n* = 561) were administered identical assessments as the intervention group, were informed of the results, and were referred to appropriate medical or social service providers in the community.	Educational intervention with specific management of risk factors in intervention group.	The efficacy of individual level risk-reduction efforts designed to prevent preterm/repeat preterm (describe low literacy as their main outcome).	Prevalence of low HL 22.5% (*n* = 106)	Not specified	Women on Medicaid or without insurance were more likely than women with private insurance to have low HL (26.2% vs. 14.1%)	Acceptance rate and participation rate	Acceptance rate (68.9%; *n* = 73) and participation rate (40.2%, *n* = 43)	Not specified
Yee et al., 2014 [34]	CG (*n* = 75): receiving standard of care counselling. IG (*n* = 75): receiving standard of care counselling and an interactive patient education tool for prenatal screening and diagnosis tests.	Interactive education tool.	Determine whether an interactive computer program could improve patient knowledge regarding genetic screening and diagnostic concepts.	% of questions correctly answered: pre 69.4 ± 14.2 post 23 days: 60.6 ± 16.0	% of questions correctly answered: pre 46.0 ± 15.2 post 23 days: 49.7 ± 18.9	pre-test *p* < 0.001 post-test *p* = 0.001			
Zite et al., 2011 [35]	Each participant was provided with a copy of either the standard (*n* = 99) or the low-literacy Medicaid-Title XIX SCF (*n* = 102) and an allocated sterilization consent form after that.	Text adaptation to HL level.	To estimate whether the Medicaid-Title XIX Sterilization Consent Form (SCF) format standard compared with low literacy is associated with women’s understanding of tubal sterilization.	77.5% of correct answers	49.0% of correct answers	*p* < 0.01 women randomized to the low-literacy Medicaid-Title XIX SCF group better understood the length of time required between signing the form and undergoing sterilization	Preference of subjects	94% (*n* = 189) preferred low-literacy Medicaid-Title XIX SCF	6% (*n* = 12) preferred Medicaid-Title XIX SCF

## Abbreviations

CA	Control arm
CG	Control group
CINAHL	Cumulative Index to Nursing and Allied Health Literature
CVI	Content validity index
CVR	Content validity ratio
HL	Health literacy
HLQ	Health Literacy Questionnaire
HLS-EU-Q47	European Health Literacy Survey Questionnaire
IA	Intervention arm
IG	Intervention group
IHLQ	Iranian Health Literacy Questionnaire
LSQ	Lifestyle Questionnaire
MeSH	Medical subjects heading
MHLAPQ	Maternal Health Literacy and Pregnancy Outcome Questionnaire
PICO	PICO question (population/intervention/comparation/outcome)
PRISMA	Preferred Reporting Items for Systematic Reviews and Meta-Analyses
PROSPERO	International prospective register of systematic reviews
RCT	Randomized clinical trial
REALM	Rapid Estimate of Adult Literacy in Medicine
RoB2	Risk of Bias tool version 2
SILS	Single Item Literacy Screener test
S-TOFHLA	Short version of the Test of Functional Health Literacy in Adults
WHO	World Health Organization
